# Nutritional status and serum zinc and selenium levels in Iranian HIV infected individuals

**DOI:** 10.1186/1471-2334-8-165

**Published:** 2008-12-09

**Authors:** H Khalili, A Soudbakhsh, M Hajiabdolbaghi, S Dashti-Khavidaki, A Poorzare, AA Saeedi, R Sharififar

**Affiliations:** 1 Department of Pharmacotherapy, School of Pharmacy, Tehran University of Medical Sciences, Tehran, Iran; 2 Department of Infectious Disease, School of Medicine, Iranian Research Center for HIV/AIDS (IRCHA), Tehran University of Medical Sciences, Tehran, Iran

## Abstract

**Background:**

Human immunodeficiency virus infected individuals are prone to malnutrition due to increased energy requirements, enteropathy and increased catabolism. Trace elements such as zinc and selenium have major role in maintaining a healthy immune system. This study was designed to evaluate the nutritional status of Iranian subjects who were newly diagnosed with human immunodeficiency virus infection and to compare serum level of zinc and selenium in these patients with those of the sex and aged match healthy subjects.

**Methods:**

After an interview and physical examination, nutritional assessment was done based on clinical and anthropometric parameters. Body mass index (normal range 18.5–27 kg/m^2 ^based on age) of less than 16, 16–16.9 and 17–18.4 kg/m^2 ^were considered as severe, moderate and mild malnutrition respectively. Serum level of zinc and selenium were measured by graphite furnace atomic absorption.

**Results:**

Severe, moderate and mild malnutrition were detected in 15%, 38% and 24% of human immunodeficiency virus infected individuals respectively. Compared with the healthy control group, serum level of zinc and selenium in the human immunodeficiency virus infected subjects were significantly lower (P = 0.01 and P = 0.02 respectively).

**Conclusion:**

Malnutrition found to be prevalent in Iranian human immunodeficiency virus infected individuals and low serum zinc and selenium levels are common in this population.

## Background

Human Immunodeficiency Virus (HIV) infection is a major health problem in the world and HIV infected individuals are vulnerable to malnutrition due to several factors including inadequate nutrient intake (anorexia, gastrointestinal complications such as nausea and vomiting, oral and esophageal sores), nutrient loss (malabsorption and/or diarrhea), metabolic alteration (increased protein turn over and changes in fatty acid metabolism), and drug-nutrient interactions [1,2].

Although malnutrition is more frequent at the end of HIV infection course, it can also occur at the onset of the disease, before sever immunosuppression [3].

Malnutrition and HIV infection can deteriorate immune system function including decline in CD4 lymphocyte count and delayed type immune reactions [2].

Functional status and survival of HIV-infected patients are affected by their nutritional conditions [4]. The critical role of nutritional support and highly active anti-retroviral therapy (HAART) in HIV-infected individuals has been approved. American Dietetic Association recommends nutritional support as a part of the care provided to HIV-infected patients [5].

Trace elements especially zinc (Zn) and selenium (Se) are important for maintaining a healthy immune system. Zinc deficiency can declines T cells generation and depresses humoral and cell-mediated immunity [6,7]. Selenium deficiency also has several medical implications including impaired immune response [8]. Taylor et al proposed a role for Se modification both in-vitro and in-vivo in HIV infection [9]. The main route of HIV transmission in IRAN is via injection drug use (IDU) and there is no data about nutritional status among this population. The goals of this study were 1) to evaluate nutritional status of newly diagnosed Iranian HIV-infected individuals and 2) to compare the serum Zn and Se levels in these patients with those of healthy individuals.

## Method

This study is a one-year cross-sectional, descriptive analytic survey conducted at Iranian Referral HIV/AIDS Research Center affiliated to Tehran University of Medical Sciences. This center is supported by Iranian Ministry of Health and Medical Education and provides free services such as Para-clinical, clinical and consultation for each volunteer who may be at risk of infection by HIV or any other sexually transmitted disease. The study group included newly diagnosed HIV infected adult males whose infection was confirmed with the anti-HIV antibody tests (ELISA and Western-blot). The control subjects were age matched healthy males related to HIV infected individuals (who accompanied HIV infected patients), without any medical problem at the time of the study or history of any chronic disease and with negative anti-HIV antibody test.

The study protocol was approved by the institutional review board and all patients provided written consent form.

During patient's interview, demographic data including social, behavioral and medical history were collected in the designed forms. Nutritional status of each patient was assessed using anthropometric parameters. Body weight was determined to the nearest 0.1 kilogram (kg) using adult balance and standing height was determined to the nearest one centimeter (Cm). Body Mass Index (BMI) was calculated using the following formula: BMI = Body Weight (kg) divided by [Height (m)]^2^. BMI (normal range 18.5–27 kg/m^2^based on age) of less than 16, 16–16.9 and 17–18.4 kg/m^2 ^were considered as severe, moderate and mild malnutrition respectively [10]. All patients were asked about body weight changes during past six months. Up to 10% body weight loss was considered significant and more than 10% body weight loss was considered severe weight loss [11]. According to definition of Center of Disease Control and Prevention (CDC), wasting syndrome was defined as an involuntary weight loss of greater than 10% of baseline body weight during the past 12 months or at least 5% loss of body weight during the past six months [12].

Clinical assessments including medical history and physical examination were conducted to identify any sign or cause of malnutrition. Physical appearance, opportunistic infections, diarrhea, symptoms of gastrointestinal distress such as nausea and vomiting, medications, use of herbal supplements and functional status were considered in the clinical assessment. Hepatitis B surface antigen (HBsAg) was detected by second generation enzyme linked immunoassay kit (ELISA), Diasorin Italy and third generation ELISA kit, DRG, Germany was used for HCV-Ab detection.

Six milliliters (mL) of fasting blood sample was collected from all HIV-infected and healthy subjects under trace elements free condition. Blood samples were centrifuged at 3000 g for five minutes. The collected serum was stored at -70°C until analysis. All glassware and bottles used for separation of serum and further analysis were previously soaked in 10% nitric acid and rinsed thoroughly with de-ionized water. Serum Zn and Se levels were measured using atomic absorption spectrophotometer (Atomic absorption spectrophotometer Shimadzu, AA-680, Japan). Samples and standards concentrations were read in duplicate.

For this study, Zn deficiency was defined as a serum level < 67 mcg/dL, using the cut-off referenced by Bender and Bender for normal plasma Zn (67–183 mcg/dL) [13]. Selenium deficiency was defined as a level < 85 mcg/L, which is a cut-off associated with increased mortality [14].

Data was analyzed using SPSS (Chicago, IL, USA) software, version 11.5. Normal distribution of data were assessed using Kolmogorov-Smirnov test and independent sample t-test was used to compare numeric variables such as age, weight, high, serum albumin, serum Zn and Se levels between HIV-infected patients and healthy subjects. Differences between serum Zn and Se concentrations from recommended cutoffs were evaluated by one-sample t-test. ANOVA was used to compare serum Zn and Se concentrations between groups that were categorized based on malnutrition severity. For determination of differences between severity and prevalence of malnutrition between HIV infected individuals and healthy group, chi-square and fisher-exact test were used. Correlations between data were evaluated using pearson correlation. Descriptive statistics (cross-tabs) followed by selection of chi-square and risk were used for generation of odds ratio and confidence interval. P-values of less than 0.05 were considered as significant.

## Results

One hundred HIV-infected adult patients with the mean age of 35.4 ± 7.8 years (range: 21–45 years old) and 100 healthy individuals with the mean age of 32.4 ± 7.8 years (range of 20–43 years old) completed this study. Table 1 shows socioeconomic status and past medical history of HIV-infected patients. Comparisons of demographic characteristics (including age, weight, height, BMI) and some nutritional parameters (including serum albumin concentration, and CD4 lymphocytes count) of HIV-infected and healthy subjects are shown in table 2. None of the HIV-infected individuals express any significant past medical problem. In this study 31% of injection drug users had a history of medication consumption such as benzodiazepines, acetaminophen-codeine, tramadol, antibiotics and non-steroidal anti-inflammatory drugs and 3% of them used herbal medications. None of the HIV-infected patients had a history of vitamin or mineral supplementary consumption.

**Table 1:** Demographic data including socioeconomic status of HIV infected individuals

**Parameters**	**Number of patient (Percent)**
**Route of HIV infection**	
IDU*	63 (63)
IDU and sexual contact	9 (9)
Sexual	20 (20)
Unknown	8 (8)

Mean period of intravenous injection	2.1 ± 1.6 years

Positive prison history	29(29)

Substances injected (in IDU group)	
Heroine	64 (89)
Others (cocaine, buprenorphine, benzodiazepines, barbiturates)	8 (11)

Smoking	61 (61)

Alcohol	7 (7)

**Marital status**	
Married	47 (47)
Unmarried	53 (53)

**Employment condition**	
Employed	37 (37)
Unemployed	63 (63)

Mean monthly Income	500000 Rials (50 $)

**Education**	
0–10 years	83 (83)
> 10 years	17 (17)

Homeless	21 (21)
With family	79 (79)

HBs-Ag positive	11 (11)

Anti-HCV Ab positive	38 (38)

*IDU: Injection Drug Use

**Table 2:** The study groups baseline characteristics

**Parameters**	**HIV infected Patients**	**Healthy group**	**P-value**
Number of subjects	100	100	-
Age (year) (mean ± SD)	35.4 ± 7.8	32.4 ± 7.8	0.21
Weight (Kg) (mean ± SD)	51.0 ± 8.4	72.2 ± 12.9	0.04
Height (Cm) (mean ± SD)	169.9 ± 10.1	171.0 ± 9.1	0.43
BMI^1 ^(kg/m^2^) (mean ± SD)	17.3 ± 2.3	24.2 ± 3.0	0.03
IBW^2 ^(kg) (mean ± SD)	68.3 ± 7.2	71.0 ± 10.2	0.54
Serum Albumin (g/dL) (mean ± SD)	2.6 ± 1.4	3.6 ± 1.7	0.03
CD4 (Cell/mm^3^) (mean ± SD)	253 ± 50	1120 ± 210	0.01

1-Body Mass Index.

2-Ideal Body Weight

Most of the HIV infected individuals were pale, 60% of them were anemic (serum hemoglobin level of less than 12 g/dL) and all of them had some degree of temporal atrophy. Gastrointestinal symptoms including nauseas, vomiting, diarrhea and decreased appetite were observed in 9% of patients.

Significant and severe recent weight losses were detected in 7% and 5% of patients respectively. Based on CDC definition, 12% of the patients had wasting syndrome.

Serum albumin level was significantly lower in HIV-infected patients compared with healthy subjects (P = 0.03). Seventeen percent of patients had serum albumin level of less than 2.5 g/dL. Additionally, serum albumin concentration was significantly lower in HIV-infected subjects who suffered wasting syndrome than the HIV-positive individuals without wasting (2.1 g/dL versus 2.7 g/dL; P = 0.01). There was positive correlation between patients' serum albumin level and CD4 lymphocyte count (r = 0.9, P = 0.003). Additionally individuals with severe malnutrition had a significant lower CD4 counts in comparison with individuals with normal, mild, or moderate malnutrition. (P = 0.004).

Table 3 compares the prevalence of different types of malnutrition between HIV-infected individuals and healthy subjects. Moderate malnutrition was the most prevalent type of malnutrition in HIV-infected individuals (observed in 38% of patients) followed by mild malnutrition (observed in 24% of the patients).

**Table 3:** Nutritional status* of the study populations

**Category**	**HIV infected group (percent)**	**Healthy group (percent)**	**P value**
Mild malnutrition	24 (24)	15 (15)	0.01
Moderate malnutrition	38 (38)	0	< 0.001
Severe malnutrition	15 (15)	0	< 0.001
Normal	23 (23)	85 (85)	0.006

* Nutritional status was calculated based on individual's BMI. BMI (normal range 18.5–27 kg/m^2 ^based on age) of less than 16, 16–16.9 and 17–18.4 kg/m^2 ^were considered as severe, moderate and mild malnutrition respectively.

Serum level of Zn and Se among HIV-infected and healthy subjects are shown in table 4. As can be seen serum level of Zn and Se were significantly lower in HIV-infected patients (P = 0.01, P = 0.02 respectively). Mean concentrations of Zn and Se were significantly lower than recommended cutoffs (p = 0.01 and p = 0.03 respectively). In addition those patients who may have been infected by used syringe had significant lower serum Zn (32.4 ± 10.6 vs. 67.2 ± 14.3 mcg/dL) and Se (55.8 ± 14.6 vs. 84.1 ± 9.9 mcg/L) concentrations compared with those who were probably infected via sexual contact (P < 0.001 for both comparisons).

**Table 4:** Mean ± SD of serum Zn and Se concentrations and prevalence of deficiency in HIV infected and healthy individuals

**Parameter**	**HIV infected group**	**Healthy group**	**P value**
Zn concentration (mcg/dl)	51.7 ± 14.2	82.9 ± 14.8	0.01
Se concentration (mcg/l)	66.4 ± 11.2	91.7 ± 11.9	0.02
Number of individuals with Zn deficiency (percent)	65 (65)	8(8)	< 0.001
Number of individuals with Se deficiency (percent)	38 (38)	2 (2)	< 0.001

Prevalence of Zn and Se deficiency of the HIV infected individuals and healthy persons also are shown in Table 4. As presented in this Table, 65% and 38% of HIV infected individuals had Zn and Se deficiency respectively.

Table 5 compares serum Zn and Se levels in patients with various severity of malnutrition.

**Table 5:** Serum Zn and Se concentrations in HIV infected individuals based on severity of malnutrition*

**Group**	**Zn (mcg/dL)**	**Se (mcg/L)**
Mild malnutrition	66.4 ± 10.7	85.9 ± 12.4
Moderate malnutrition	40.2 ± 11.0	58.3 ± 10.3
Severe malnutrition	33.3 ± 9.6	50.4 ± 13.2
Normal	77.2 ± 16.2	89.5 ± 11.6
P value	0.03	0.04

* Severity of malnutrition was determined based on individual's BMI. BMI (normal range 18.5–27 kg/m^2 ^based on age) of less than 16, 16–16.9 and 17–18.4 kg/m^2 ^were considered as severe, moderate and mild malnutrition respectively.

Patients with moderate malnutrition had significant lower serum Zn and Se levels than non-depleted patients. Severe malnutrition was a risk factor for Zn deficiency (Odds Ratio = 2.3 (95% confidence interval = 1.2–4.5 and P = 0.001).

## Discussion and conclusion

In this study 77% of newly diagnosed HIV-infected patients who were not in advanced phase of the disease were evaluated and founded to have some degree of malnutrition.

Malnutrition is a significant clinical problem in HIV-infected individuals. In this population, wasting has been associated with disease progression and increased mortality [15].

Although malnutrition is usually encountered at the advanced phase or end of the HIV-infection course, however, as seen in our study it may also occur in the first stages of the HIV-infection as well [16].

According to the last report of Iranian Diseases Prevention and Control Center, 94.3% of Iranian HIV infected individuals are male [17] and consequently most referred patients to our center were male, so the present study was designed for this sex group.

As anthropometric measurements provide inexpensive and non invasive method to evaluate nutritional status [18], these parameters were used in this study to evaluate nutritional status of HIV-infected individuals.

The results of this study showed moderate to severe malnutrition in 53% of the HIV-infected subjects. Contrary to other studies which stated sexual contact as the main route of HIV infection [19,20], the most prevalent route of HIV infection in our patients was IDU. Moderate to severe malnutrition were detected in 68% of this main sub-group of the patents that is comparable with the findings of Nazrul Islam et al who reported mild to severe malnutrition in more than 60% of their patients who were drug addicts [21]. Due to educational, socioeconomic and behavioral characteristics, addicted persons especially injection drug users are more vulnerable to malnutrition [21]. Most of the injection drug users in present study had less than 10 years education and were unemployed, unmarried and smoker with an insufficient monthly income. Also some of them were homeless and alcoholic.

Although it was reported that there is not correlation between body weight loss and level of immunosuppression [16], the results of this study showed significant reverse relationship between severity of weight loss and serum CD4 lymphocyte count that is compatible with the findings of Rivera et al [22].

As reported in another study [23], wasting syndrome was present in about 12% of the patients. During wasting in HIV infection, the body tries to compensate energy from available sources such as visceral proteins [24]. Although normal serum albumin concentration has been reported in HIV-infected patients [5], serum albumin levels in our patients were significantly lower than those of the healthy subjects. Also the patients with wasting syndrome had significant lower serum albumin than the patients without wasting.

Similar to the findings of Koch et al [25], Graham et al [26] and Zamrzly et al [27], serum levels of Zn and Se in our HIV-infected individuals were significantly lower than the healthy group.

This is the first study about nutritional status of Iranian HIV infected individuals and there are some limitations to our study. The first one is that the cross sectional design of the study limits our conclusions and does not provide information about effects of nutritional status on disease progression and outcome of HIV infected individuals. We have started a controlled clinical trial to assess effects of nutritional support on disease progression and outcome of HIV infected patients. In another hand we know that serum Zn and Se levels may not be the best indicator of total stores for these trace elements in the body and cellular concentrations or related enzymes activity may be more reliable.

Based on World Health Organization nutritional recommendations for HIV infected persons, adequate nutrition is critical for health and survival for all subjects regardless of HIV infection condition [28]. Following presentation of the study results, nutritional assessment is a part of clinical assessment of HIV infected individuals. Patients and their family have counselling about maintain adequate healthy diet and nutrition care in our centre. Also it is emphasized that these patients should be taking daily recommended of relevant micronutrients through diet or supplements.

In conclusion malnutrition and serum Zn and Se deficiency are common in Iranian HIV-infected patients and early evaluation of nutritional status of these subjects and providing appropriate nutritional support and mineral supplementation along with the specific anti-retroviral treatment are recommended.

## Competing interests

The authors declare that they have no competing interests.

## Authors' contributions

KH Main investigator (Leader of this research team). SA, HM Infectious disease specialist participating in the fallow-up and management of HIV infected individuals referred to Iranian HIV/AIDS Research Center. DKS Preparing of manuscript. PA Measurement of serum Zn and Se concentration. SAA Nutritional assessment. SR Patients interview.

## Pre-publication history

The pre-publication history for this paper can be accessed here:


